# *De novo*-based transcriptome profiling of male-sterile and fertile watermelon lines

**DOI:** 10.1371/journal.pone.0187147

**Published:** 2017-11-02

**Authors:** Sun-Ju Rhee, Taehyung Kwon, Minseok Seo, Yoon Jeong Jang, Tae Yong Sim, Seoae Cho, Sang-Wook Han, Gung Pyo Lee

**Affiliations:** 1 Department of Integrative Plant Science, Chung-Ang University, Ansung, Republic of Korea; 2 Department of Agricultural Biotechnology and Research Institute of Agriculture and Life Sciences, Seoul National University, Seoul, Republic of Korea; 3 Interdisciplinary Program in Bioinformatics, Seoul National University, Kwan-ak Gu, Seoul, Republic of Korea; 4 CHO&KIM Genomics, C-1008, H Business Park, 26, Beobwon-ro 9-gil, Songpa-gu, Seoul, Republic of Korea; Chungnam National University, REPUBLIC OF KOREA

## Abstract

The whole-genome sequence of watermelon (*Citrullus lanatus* (Thunb.) Matsum. & Nakai), a valuable horticultural crop worldwide, was released in 2013. Here, we compared a *de novo*-based approach (DBA) to a reference-based approach (RBA) using RNA-seq data, to aid in efforts to improve the annotation of the watermelon reference genome and to obtain biological insight into male-sterility in watermelon. We applied these techniques to available data from two watermelon lines: the male-sterile line DAH3615-MS and the male-fertile line DAH3615. Using DBA, we newly annotated 855 watermelon transcripts, and found gene functional clusters predicted to be related to stimulus responses, nucleic acid binding, transmembrane transport, homeostasis, and Golgi/vesicles. Among the DBA-annotated transcripts, 138 *de novo*-exclusive differentially-expressed genes (DEDEGs) related to male sterility were detected. Out of 33 randomly selected newly annotated transcripts and DEDEGs, 32 were validated by RT-qPCR. This study demonstrates the usefulness and reliability of the *de novo* transcriptome assembly in watermelon, and provides new insights for researchers exploring transcriptional blueprints with regard to the male sterility.

## Introduction

Watermelon [*Citrullus lanatus* (Thunb.) Matsum. & Nakai], a member of the *Cucurbitaceae* family, is an important crop worldwide, with annual production of approximately 110 million tons in 2013 (FAO, http://faostat.fao.org/). The first reference genome sequence of the East Asian watermelon was released in 2013 [1], based on next-generation sequencing (NGS) techniques. According to the genome announcement, watermelon has a diploid genome (2n = 2x = 22) of ~425 Mb, with 11 chromosomes and 23 440 transcripts. Completion of the reference genome has allowed members of the *Cucurbitaceae* to be analyzed using RNA-seq. Two common RNA-seq assembly methods are widely used: *de novo*-based approach (DBA) and reference-based approach (RBA) [2–4]. Although both approaches can be applied to transcriptome studies, they are selectively employed under different purposes and conditions, and often generate distinct results.

Since the process of RBA is more efficient than that of DBA, RBA is generally preferred when there is a well-established reference genome. On the other hand, use of DBA is unavoidable in efforts to identify transcripts in the absence of a reference genome. DBA can be also applied to incomplete reference genomes, for example in unknown species, because it is flexible to spliced transcription sites or unexpected structural variations [5, 6]. Novel transcripts may also be discovered by DBA, when reliable follow-up validation is performed. Although DBA is one of the best ways to identify transcripts without a reference genome, it carries a high computational burden and is prone to problems with uneven sequencing coverage derived from differential expression of genes, chimeric transcripts, and repeated sequences [7].

In view of the pros and cons of RBA and DBA, both approaches should be considered for RNA-seq analysis with reference genomes. The reference genomes of most non-model organisms, including watermelon, often exhibit missing expressed genes, trans-spliced genes, assembly errors, and deletions [8–10]. As the accuracy of RBA results depend on the completeness of the reference genome, the completeness of the reference genome used for RBA must be considered beforehand. The watermelon reference genome was recently published and watermelon transcriptome studies have employed RBA using it [11–17]. However, the draft version of the reference genome has not been updated yet, and thus, the reference genome as well as watermelon genome analysis should be continuously reviewed and developed by diverse genome research. To supplement the results of the previous RBA study [13], and given the advantages of DBA for an incomplete reference genome, we reasoned that it would be worthwhile to use DBA to improve transcriptomic analysis in watermelon.

In the crop industry, male sterility is an important trait for hybrid watermelon breeding as it renders emasculation unnecessary. There are three types of male sterility: cytoplasmic male sterility (CMS), genic male sterility (GMS), and cytoplasmic genic male sterility (CGMS) [18]. CMS is maternally inherited and controlled by the mitochondrial or plastid genome, GMS is inherited via the nuclear genome, and CGMS is induced by interaction of the mitochondria and nucleus when the restoration of fertility genes influences the CMS system.

Five watermelon male-sterile mutants have been reported to date, such as glabrous male-sterile (*gms*) [19–21] and male-sterile dwarf (*ms-dw*) [22], *ms-1* [23], *ms-2* [24], and *ms-3* [25]. Although several attempts have been made to identify the genetic mechanisms underlying male sterility [19, 21–26], only one of these was a transcriptome study, and it was based on RBA such that unannotated transcripts were unable to be characterized [13].

In this study, we used DBA to re-analyze RNA-seq data from our previous RBA study, as a complementary approach to gain perspectives on gene annotation and to detect novel transcripts related to male sterility in watermelon. We identified several previously unreported transcripts including *de novo*-exclusive differentially-expressed genes (DEDEGs) from the comparison between RBA and DBA. We also characterized the functional networks between those newly annotated transcripts to provide an outline of the undiscovered transcriptome. Finally, we successfully validated the presence and differential expression of novel transcripts through RT-qPCR, demonstrating the efficacy and the legitimacy of DBA in complementing RBA to analyze the underpinnings of male sterility in watermelon.

## Materials and methods

### Watermelon samples and RNA-seq experiments

The two watermelon lines employed in this study were the genic male sterile (GMS) line DAH3615-MS (MS), *msms*, and the fertile, near-isogenic line DAH3615 (MF), *Msms*, which is derived from the *ms-1* Chinese male sterile line [23]. The plant materials and total RNA isolation for production of raw RNA-seq data in this study are described in our previous report [13]. Raw RNA-seq data used in this article are available in the GEO database under accession number GSE69073.

### DBA and RBA data processing

For DBA, prior to assembly, Illumina adapter sequences were removed using Trimmomatic [27]. Clean transcripts were assembled using Trinity (r20140717) [28]. After generating transcript contigs, RNA-seq reads were mapped to the constructed transcriptome reference using Bowtie 2 [29], and RSEM [30] to align and quantify reads. Isoform and gene count matrices were generated using *abundance_estimates_to_matrix*.*pl* implemented in Trinity. Finally, contigs were annotated using Trinotate (r20140708) (https://trinotate.github.io/), and only plant-originated transcripts (*Viridiplantae*) were used for downstream analyses. Among those isoform transcripts, the longest was selected as the representative sequence for each gene.

To compare DBA and RBA, RBA results from a previous study [13] were used. The watermelon reference genome (cv. 97103) version 1 from the Cucurbit Genomics Database [1] was employed. In addition, Trimmomatic [27], Tophat2 [31], and HTSeq-count [32] were used to quantify the abundance of mapped reads and to annotate watermelon genes. UniProtKB gene identification was used to compare gene lists between DBA and RBA.

### Statistical analysis to identify DEGs in MF and MS

Considering our 2 x 2 factorial experimental design, analysis of variance (ANOVA) was used for RNA-seq and qPCR analysis. First, a negative binomial-assumed two-way analysis of deviance (ANODEV) model was employed for RNA-seq analysis as follows:

$$log({E(Expression}_{ij}))=\mu +{breed}_{i}+{tissue}_{j}$$ (Eq 1)

where *i* = MF and MS lines, *j* = floral bud and flower.

The effect of breeding line (MF or MS) and tissue on the detection of male-sterility-related genes was tested statistically using *edgeR* implemented in R [33]. Significance cutoff was used at the FDR adjusted *P*-value ≤ 0.01. Likewise, a two-way ANOVA model was employed for the qPCR experiment because the value of Δ*ct* is commonly used to derive relative gene expression–usually satisfied according to the assumption of a normal distribution. The statistical model used was as follows:

$${-\Delta ct}_{ij}=\mu +{breed}_{i}+{tissue}_{j}+\varepsilon_{ij},\varepsilon_{ij}∼N(0,\sigma^{2})$$ (Eq 2)

The value of Δ*ct* was calculated based on the difference of time it took to reach the threshold between control and targeted genes, which is negatively correlated with RNA-seq gene expression. To make the direction of Δ*ct* represent gene expression, the −Δ*ct* value was employed in the analysis.

### Functional terms and network analysis of significantly enriched terms

The Database for Analysis, Validation, and Integrated Discovery (DAVID) was used to characterize specific gene lists [34, 35]. Three categories of functional terms from the GO database were employed: BP, MF, and CC. In addition, since most already-annotated genes derived from *Arabidopsis thaliana*, the *Arabidopsis* gene annotation was used as a background. Significance was considered at a *P*-value ≤ 0.001 for newly annotated transcripts and a *P*-value ≤ 0.01 for DEDEGs. Generally, transcripts are described in diverse biological terms; therefore, functional terms can be classified based on M:N relationships. Significantly enriched terms were first combined, then a gene association matrix was generated (Terms x Genes). Binary values were used in this matrix, i.e., 0 means that a gene does not have a specific function, and 1 means that a gene does have a specific function. Using this matrix, correlation-based network analysis was conducted and the FDR adjusted *P*-value <0.01 was considered as a significant relationship. Finally, identified relationships were visualized in network format using *qgraph* package implemented in R [36]. Spring layout was also used to classify similar terms based on the strength of their connections.

### RT-qPCR for technical validation

Primers for a total of 33 randomly selected candidates [14] newly annotated transcripts (Tables G and H in S1 File) and 19 DEDEGs (Tables I and J in S1 File)] were designed using Primer3 [37]. Three biologically replicated samples of floral buds and mature flowers were collected from individual MF and MS plants, respectively. cDNA was synthesized by SuperScript RTaseIII (Thermo Fisher Scientific, USA) and oligo (dT)_15_ using 1 μg total RNA isolated from each sample. Watermelon 18S rRNA was used as an internal control to normalize mRNA. Reagents used for qPCR were 10 μl PCR pre-mix, 1 μl evergreen fluorescence dye (SolGent, Korea), 1 μl cDNA, and 500 nM of each primer (except for the 18S control experiment in which 250 nM of each primer was used). PCR conditions were as follows: 95°C for 12 min, 40 cycles at 95°C for 10 s, and 60°C for 30 s.

## Results

### Summary of sequencing and *de novo* transcriptome assembly statistics

We previously produced raw RNA-seq data for four watermelon samples: flower and floral bud tissue samples of male-sterile and male-fertile lines (2 breeding lines × 2 tissues) [13]. The four samples contained read numbers ranging from 25 299 088 to 29 490 814. Approximately 80% of the total reads in all samples met Q30 quality control criteria (Table A in S1 File). Here, *de novo* assembly was performed after removing poor-quality reads and adapter sequences. A total of 50 581 312 reads were used to define transcripts, resulting in 138 811 transcripts (Table B in S1 File). The average length of assembled transcripts was 1100 bp and the N50 was 2032 bp. For transcripts containing multiple isoforms that differed because of splicing events, the longest isoform was chosen to represent each gene. In all, 94 496 candidate genes were assembled; the average length of assembled genes was 773 bp and the N50 was 1327 bp (Table B in S1 File).

### Gene annotation and discovery of novel genes

The 94 496 assembled transcripts were queried against the Swiss-Prot database using BLASTP and BLASTX [38]. Prior to further analyses, we selected only plant-originated watermelon transcripts by filtering annotated genes from species belonging to the kingdom *Viridiplantae*; 11 072 and 14 398 plant-originated transcripts were annotated in DBA by BLASTP and BLASTX, respectively (E-value ≤ 10^−5^). The BLASTP search originally annotated fewer transcripts (11 072) than the BLASTX annotation (14 398), but removal of duplicated transcripts based on UniProtKB ID reduced the difference between the two BLAST annotations (BLASTP, 7135; BLASTX, 8045).

**Fig 1:**
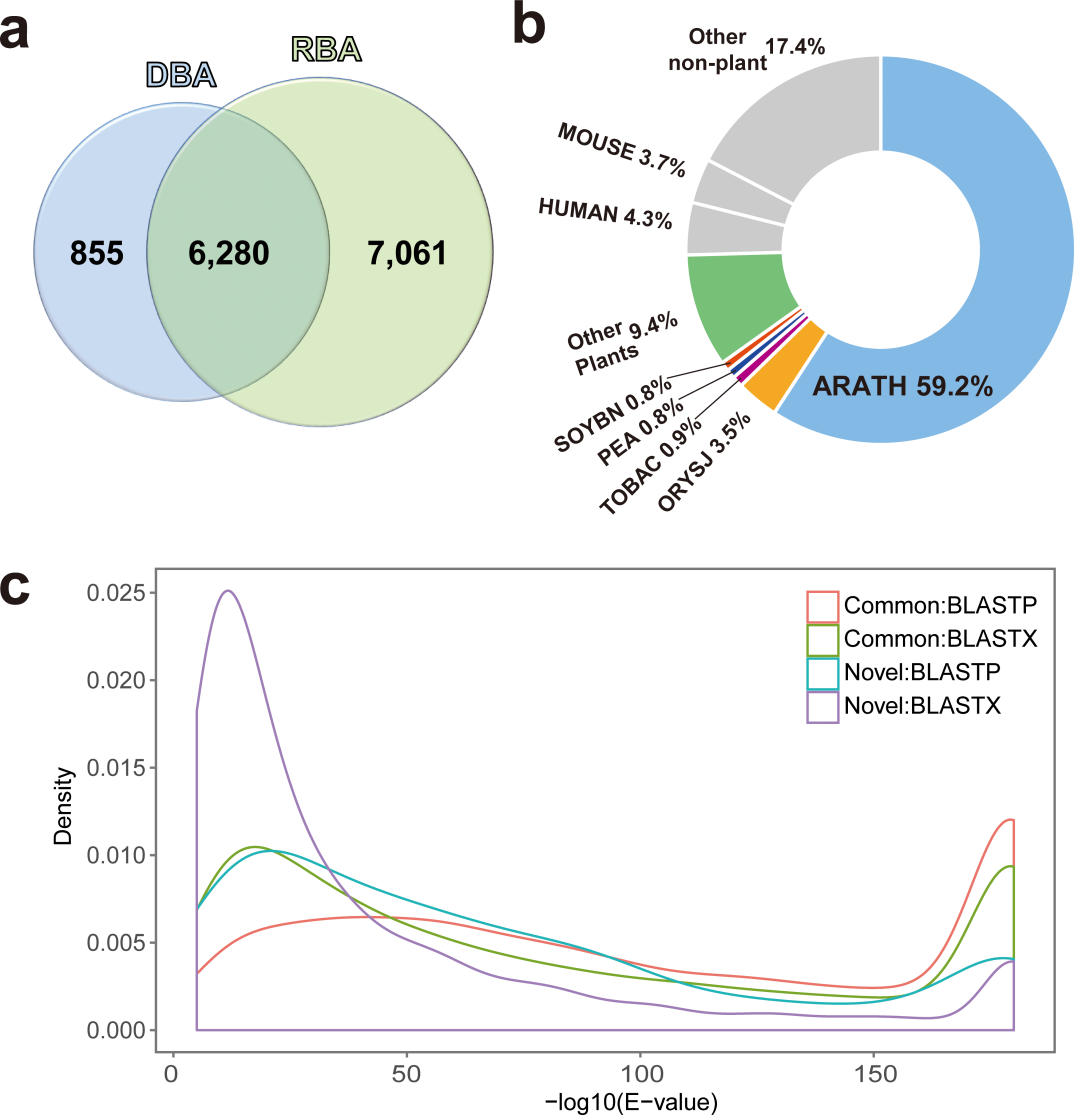
Summary of DBA annotation. (a) Venn diagram of DBA and RBA annotations using BLASTP against the Swiss-Prot database. Numbers represent deduplicated UniProtKB IDs included in each subset. (b) Pie chart denotes the origin of the sequences upon which transcript annotations were based. The top five plant species were *Arabidopsis thaliana* (ARATH), *Oryza sativa subsp*. *japonica* (ORYSJ), *Nicotiana tabacum* (TOBAC), *Pisum sativum* (PEA) and *Glycine max* (SOYBN). Gray chart represents non-plant originating species: *Homo sapiens* (HUMAN) and *Mus musculus* (MOUSE). (c) The density of E-values in common and novel transcripts (derived from BLASTP and BLASTX) was investigated. The x and y axes represent–log10 scaled E-value and density, respectively. An E-value of 0 was converted to the second lowest E-value (1×e^-180^).

To detect novel transcripts, we compared the annotations derived from DBA and RBA after removing duplicated annotations using UniProtKB ID [39]. BLASTP analysis suggested that 6280 of 7135 nonduplicated transcripts (88.0%) were commonly identified between DBA and RBA, and detected 855 putative novel genes (1132 transcripts with duplicated UniprotKB ID) (Fig 1A). A similar pattern was also observed in the BLASTX annotations (S1 Fig): a large proportion of transcripts (6673, 82.9%) were common between both DBA and RBA, and 1372 genes were newly identified by DBA.

Despite the difference in employment of query sequence (BLASTP, predicted protein coding sequence; BLASTX, conceptual translation of sequence), the concordance between BLASTP and BLASTX analysis demonstrates robust annotation. Although the number of transcripts presented was larger in the BLASTX results (14 398) than in the BLASTP results (11 072), the proportion of unique gene annotation was higher with BLASTP (64.4%) than BLASTX (55.9%), as was the proportion of DBA-RBA common gene annotation (88.0% in BLASTP versus 82.9% in BLASTX). The majority (10 888 of 11 072) of *de novo* assembled contigs annotated in BLASTP were also annotated in BLASTX. Additionally, BLASTP annotation appeared to represent a relatively conservative annotation result in terms of its skewness towards lower E-values (Fig 1C). For this reason, we chose the BLASTP annotation for the downstream analyses. In all, 11 072 transcripts (7135 UniProtKB identified genes) were annotated based on BLASTP from DBA using the DAH3615 watermelon lines. Investigation of the annotation sources of those transcripts (Fig 1B) revealed that a majority of transcripts (74.6%) were most closely related to genes from plants, especially from *Arabidopsis thaliana* (59.2%).

### Functional network analysis of novel transcripts

**Fig 2:**
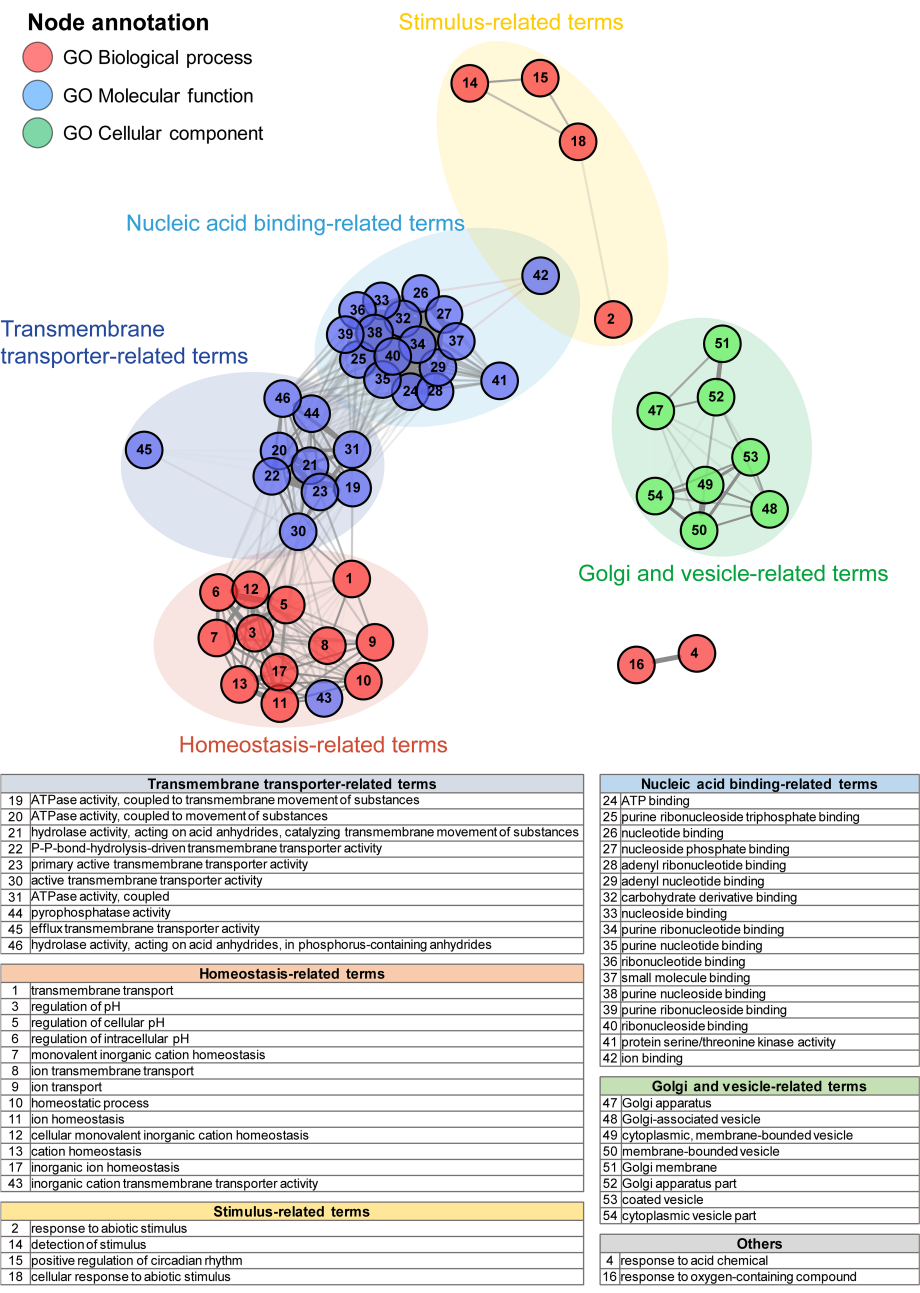
Functional GO term analysis for newly annotated transcripts. A total of 855 newly annotated transcripts were used in functional enrichment analysis. Significantly enriched GO terms (enrichment test *P*-value ≤ 0.001) were visualized in network format to cluster similar terms. Each node represents significantly enriched GO terms across three subcategories; biological process (BP), molecular function (MF), and cellular component (CC). The strength of edges depends on the correlation (only significantly correlated relationships are represented; correlation test FDR adjusted *P*-value < 0.01). Assignment of node location was determined according to centrality and numbers of related nodes. Five representative clusters were highlighted as colored circles and numbered GO terms of each cluster were shown in included tables.

To investigate the functional features of newly annotated transcripts, we performed enrichment analysis of functional terms based on three gene ontology (GO) sub-categories: ‘biological process’ (BP), ‘molecular function’ (MF), and ‘cellular component’ (CC). Eighteen, 28, and 8 functional terms were significantly enriched in BP, MF, and CC, respectively (enrichment test *P-*value ≤ 0.001, Tables C-E in S1 File). As similar biological terms were repeatedly detected across the three GO categories, we conducted network analysis of functional terms to classify analogous terms. This revealed five large clusters grouped as transmembrane transporter, homeostasis, stimulus, nucleic acid binding, and Golgi and vesicles (Fig 2). Three of the five clusters, nucleic acid binding-related, transmembrane transporter-related and homeostasis-related terms, were significantly related (in a correlation test based on a gene association matrix, FDR adjusted *P-*value ≤ 0.01), whereas two clusters, stimulus-related and Golgi and vesicle-related clusters, were independently observed. In three highly correlated clusters, diverse terms related to biological functions that controls internal homeostasis against external stimulus through transmembrane ion transport signals were significantly detected together. As these significantly enriched functional terms are fundamental in plants and other organisms, these newly annotated transcripts suggest its importance on plant viability, especially on stimulus and regulation of homeostasis.

### Identification of differentially-expressed genes (DEGs) by DBA

After removing non-expressed transcripts, we statistically analyzed 10 829 BLASTP-annotated transcripts, to not only identify DEGs associated with male sterility, but also to detect any *de novo*-exclusive DEGs (DEDEGs) that might be identified as DEGs in only DBA. Two-way analysis of deviance (ANODEV) was conducted for each transcript, taking into account the existence of both sterility and tissue-type variation. After removing duplicated UniProtKB IDs, 443 DEGs (508 transcripts with duplicated UniprotKB ID) were detected between the male-fertile DAH3615 (MF) and the male-sterile DAH3615-MS (MS) lines (FDR adjusted *P-*value ≤ 0.01).

**Fig 3:**
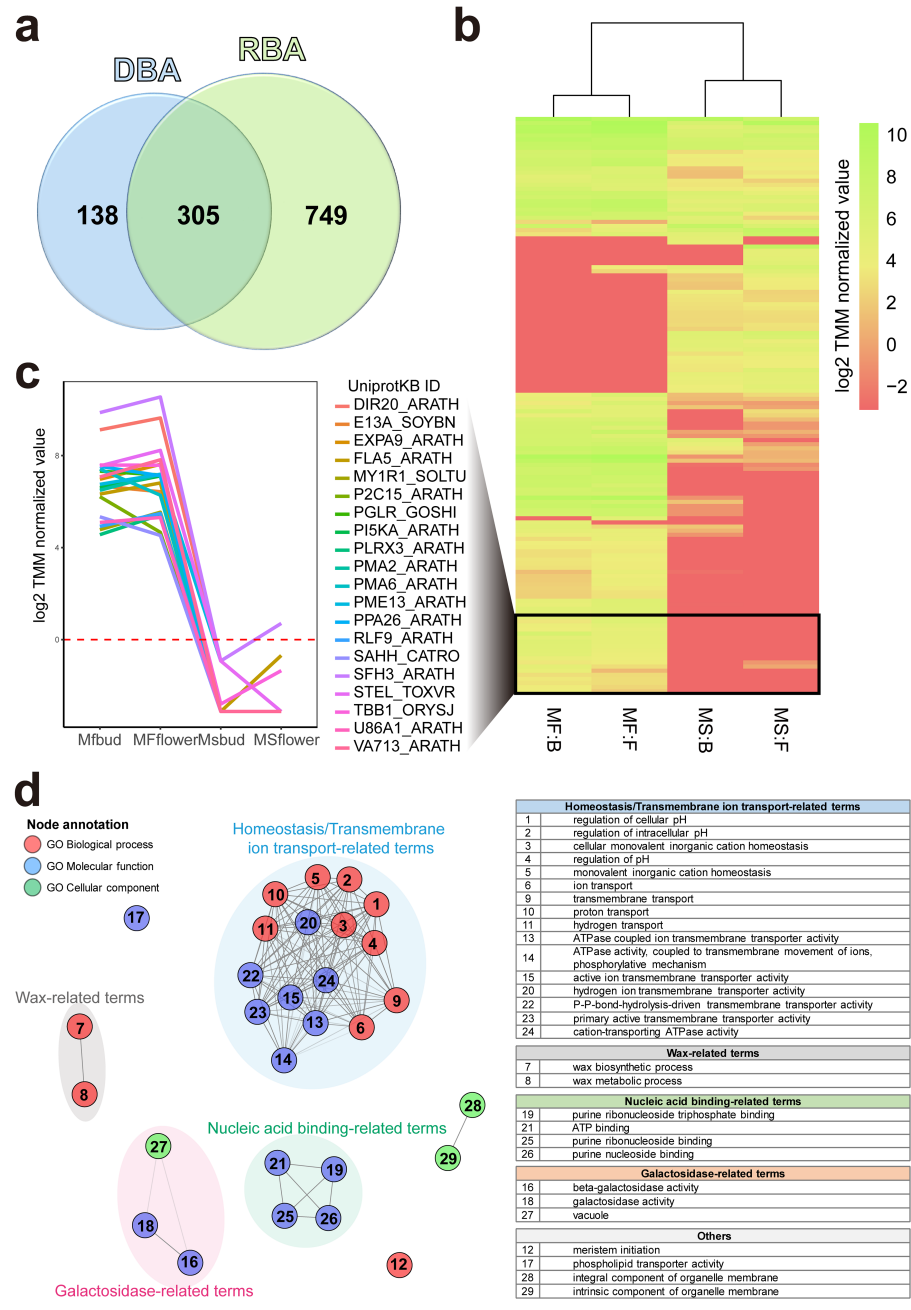
DEDEGs and their functional annotation. (a) Venn diagram comparing numbers of significant DEGs (between MF and MS lines; FDR adjusted *P*-value ≤ 0.01) in DBA and RBA, after duplicated genes were filtered out. (b) Heatmap displays expressions of 140 DEDEGs. Log2 TMM normalized values were used for gene expression. The bold rectangle represents the twenty most MF-biased DEDEGs between MF and MS, and (c) detailed expression patterns of those DEDEGs were visualized as a line plot. The red dotted line represents the log2 TMM normalized value of 0. (d) Network visualization of GO terms for 140 DEDEGs (enrichment test *P*-value ≤ 0.01). Each node indicates three categories of GO terms; biological process (BP), molecular function (MF), and cellular component (CC). Significantly correlated terms were connected to each other (correlation test; FDR adjusted *P*-value < 0.01). Four representative clusters were highlighted as colored circles and numbered GO terms of each cluster were shown in included tables.

Comparing the lists of DEGs identified via DBA and RBA, 138 nonduplicated DEGs (representing 140 transcripts) were identified as DEDEGs (Fig 3A). The gene expression pattern of these transcripts was visualized as a heatmap (Fig 3B), which revealed drastic differential expression of DEDEGs between MS and MF groups. Of these genes, 20 genes were up-regulated in MF, and showed no expression in MS (Fig 3C).

Functional enrichment analysis to identify the functional characteristics of these genes revealed significantly enriched biological terms (enrichment test *P*-value ≤0.01) across the three GO categories (Fig 3D and Table F in S1 File). Network analysis revealed four functional clusters: terms related to homeostasis/transmembrane ion transport, wax, nucleic acid binding, and galactosidase. Homeostasis, transmembrane ion transporter activity and nucleic acid binding-related functional clusters were particularly common among newly annotated transcripts (Fig 2) and DEDEGs (Fig 3D).

### Technical validation of novel transcripts and DEDEGs

Since no biological replications were used in our RNA-seq experiment, replicates were needed to validate the technique. Three biological replicates were subjected to real-time quantitative reverse transcription- polymerase chain reaction (RT-qPCR), with three technical replicates for each of four samples denoted as MF:B, MF:F, MS:B, and MS:F (B, floral bud; F, flower), for 33 randomly selected transcripts (14 newly annotated transcripts and 19 DEDEGs).

**Fig 4:**
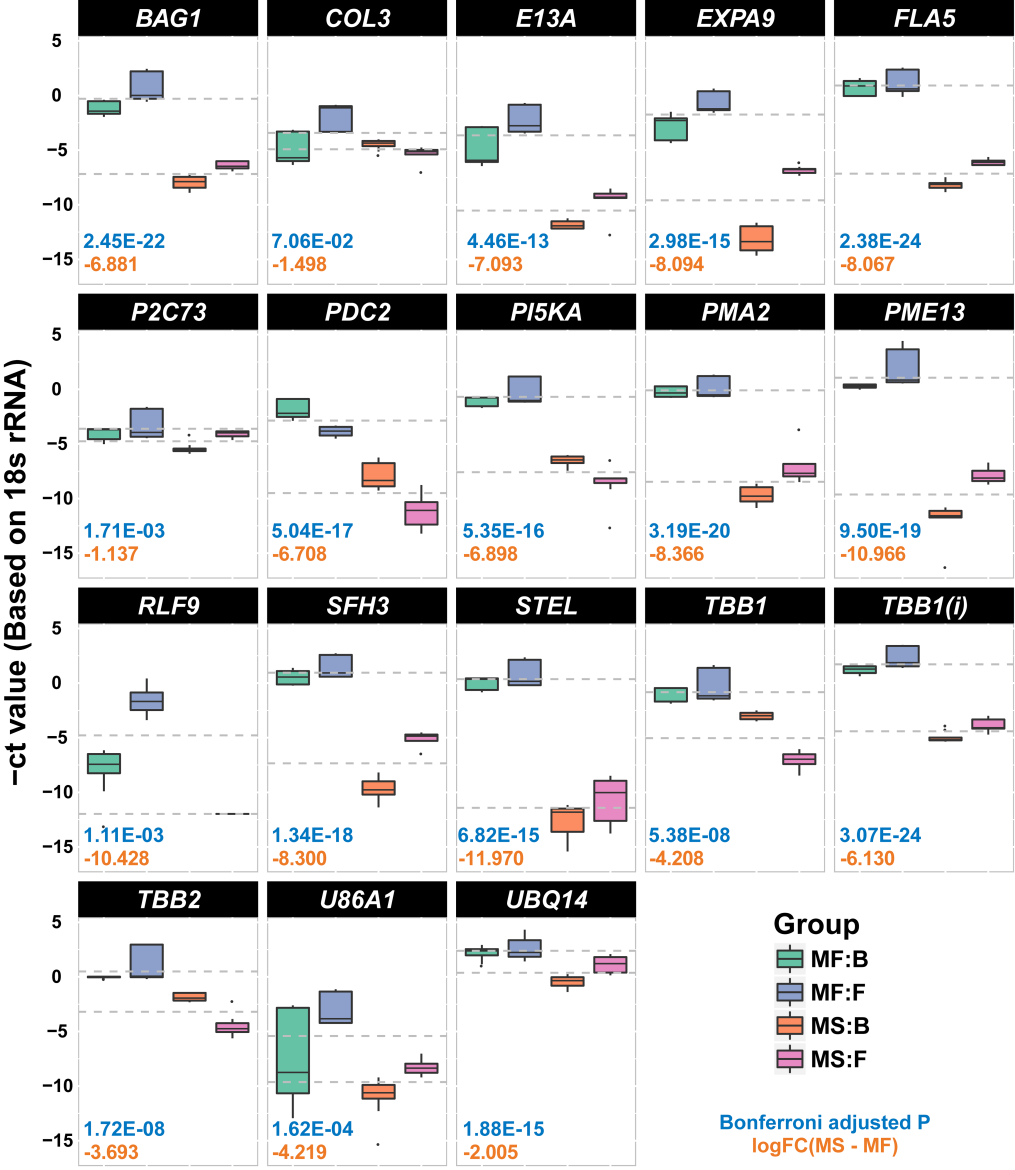
Technical validation of 19 DEDEG using RT-qPCR. Box plots were visualized with gene expression values (-ΔCt values) measured using RT-qPCR experiments to technically validate 19 significantly detected DEDEGs. The ΔCt values were successfully measured in 18 out of 19 targeted genes. The y-axis shows -ΔCt values against the control gene (18s rRNA). UniProt gene IDs are shown on top of the boxes. Bonferroni’s adjusted *P*-values are labeled as blue text and log2 fold-changes (MS–MF) are labeled as red text in each gene. Colored boxes represent each group; MF:B, MF:F, MS:B, MS:F. *(i): different transcripts but identical gene ID.

First, RT-qPCR was performed on 14 randomly selected candidates for newly annotated transcripts to investigate reliability. We presumed that the existence of the target transcripts could be demonstrated based on their relative gene expression measured against the control gene, regardless of condition. In this way, gene expression for all 14 newly annotated transcript candidates was successfully detected (S2 Fig), thus demonstrating the reliability of the discovery of novel transcripts, and supporting the use of DBA as complementary to RBA in watermelon. Next, to verify DEDEGs, 19 candidates of 138 DEDEGs (MF vs. MS, FDR adjusted *P*-value ≤ 0.01) were randomly selected for RT-qPCR validation. Two-way ANOVA was used to determine the statistical significance of differential expression of 19 DEDEG candidates. The relative gene expression of each transcript was also compared based on RT-qPCR, although one gene (*RLF9*) failed to reach the threshold. The other 18 transcripts were all significantly detected as DEGs by comparing MF and MS (Fig 4) (Bonferroni’s adjusted *P*-value ≤ 0.01). Log 2-fold change (log2FC) values indicated that all of these transcripts were highly down-regulated in MS samples, providing evidence for their fertility-biased expression and association with male sterility.

## Discussion

RNA sequencing (RNA-seq) is more cost- and time-effective than expressed sequence tag (EST), qPCR, or microarray analysis and can be used to directly construct *de novo* transcriptomes of non-model organisms [40]. Transcriptome/genome analysis through RNA-seq can be effectively accomplished through RBA when a reference genome exists, but RBA is completely dependent on the degree of completion of the reference genome. DBA, an alternative approach using *de novo* transcriptome assembly, can produce distinct results irrespective of presence or quality of a reference genome, but it requires a high degree of computation capacity. Further, RBA and DBA can be used independently or as a combined method. Reference genomes of major model organisms have been updated continuously based on follow-up research [41]. Since its release, the reference genome of watermelon has been served a crucial role in watermelon genome analyses; however, the current watermelon reference genome is only a draft version published at 2013 and has not been not updated yet. For these reasons, we applied RBA and DBA simultaneously to uncover the watermelon transcriptome and to contribute to watermelon genome analysis.

In the watermelon RNA-seq studies using RBA, different numbers of annotated genes were observed, indicating differences in transcriptome profiling, likely attributable to various factors such as experimental conditions or tissue/breed specificity [11, 12, 14]. Thus, gene annotations in diverse tissues and breeding lineages are helpful to explore such specificities via RNA-seq analysis. Based on our previous RBA study, we speculated that complementary annotation is needed to elucidate the missing part of the previous RBA, considering the relative infancy of the watermelon reference genome. The low mapping rates to the watermelon reference genome (51.0–54.7%) compared to that of *Arabidopsis thaliana* also indicate the insufficiency of RBA using the watermelon reference with DAH3615 lines, implying that application of DBA could be helpful to complement the reference-based watermelon transcriptome in terms of providing genomic information [42, 43].

Here, we compared DBA and RBA approaches on the same RNA-seq data to identify whether DBA would improve upon RBA-based annotation and provide distinct results. With regard to the low mapping rates on the reference genome, we speculated that the individual application and comparison of DBA and RBA would minimize the loss of information, and enable more straightforward observation of the watermelon transcriptome than another combined strategy, such as align-then-assemble. To minimize false positives and conservatively compare DBA with RBA, we collected only plant-derived transcripts for BLASTP annotation of the *de novo* assembled transcriptome, and discovered 855 new transcripts that represent parts of the transcriptome thus far undiscovered by RBA (Fig 1A). Since these novel findings may provide valuable information that RBA could not detect, it was necessary to validate their reliability–given that all background information is generated *ab initio*, DBA is particularly prone to the problem of false positives.

Four notable pieces of evidence support the reliability of DBA in this study. First, while 855 genes (1132 transcripts) were newly detected, 6280 (88.0%) genes from DBA were commonly identified in RBA (Fig 1A). Second, the large proportion (74.6%) of annotated transcripts were most closely related to those from plants, including *Arabidopsis thaliana* (59.2%) (Fig 1B), despite the fact that the functional annotation in the Swiss-Prot database is generally skewed towards representative mammalian organisms. Third, E-value distribution in the annotation step revealed that many annotated transcripts matched uniquely between the database and query sequences; this finding indicates that the annotated contigs were well assembled with clarity (Fig 1C). Finally, when 14 of the 855 newly identified transcripts were selected for RT-qPCR with biological replicates, gene expression could be confirmed for all transcripts (S2 Fig). This result supports the idea that the newly identified transcripts derived from DBA are bona fide. Taken together, these results show that DBA provides distinct benefits for watermelon transcriptome research; the identification of 855 novel transcripts is valuable for complementing the available RBA genomic information of watermelon.

For an overview of the possible functional properties of the 855 newly annotated watermelon transcripts, we conducted functional term network analysis. We detected five large clusters related to transmembrane transporter, homeostasis, stimulus, nucleic acid binding, and Golgi and vesicles (Fig 2 and Tables C-E in S1 File). Genes assigned to these terms serve basic but crucial roles in cellular activities of plants. Three of the five clusters, nucleic acid binding-related, transmembrane transporter-related and homeostasis-related terms, were especially highly correlated. These serve a role in one of the most important cellular processes, cell survival through regulation of homeostasis. The fact that the functional terms of these newly annotated transcripts are relevant to basic processes occurring in the plant provides evidence of the necessity for DBA in watermelon. Thus, we conclude that the use of DBA contributes useful insights and enables diverse interpretations in further watermelon research.

Of our 855 newly annotated transcripts, DBA uniquely revealed 138 transcripts that were differentially expressed between DAH3615 and DAH3615-MS; we termed these DEDEGs. A previous transcriptome study using RBA suggested the existence of biased expression between MF and MS groups [13], and indeed a similarly biased pattern was observed among our DEDEGs (Fig 3B and 3C). Considering the phenotypic differences between male sterility and fertility and the fertility biased-expression patterns observed in both RBA and DBA analyses, this observation suggests that the DEDEGs are candidates for serving roles in male sterility along with DEGs previously discovered by RBA.

The 138 DEDEGs we observed formed four functional clusters including homeostasis/ ion transporter, wax, nucleic binding, and galactosidase-related terms (Fig 3D and Table F in S1 File). These clusters are frequently observed to function in plant sterility. Ion transporters are involved in signal transduction, cell wall metabolism, and rearrangement of cytoskeletons [44]. Such transporters are enriched in pollen and involved in pollen maturation and pollen tube elongation. Notably, *Ms-cd1* mutant cabbage producing collapsed pollen showed repression of various ion transporters in floral buds [45]. Galactosidase is a cell wall modifying enzyme that is involved in microspore development [46]. The anther surface and pollen exine are composed of cutin and intra- and epi-cuticular waxes [47]. Similar to our results, wax-related genes and galactosidase-related genes have been reported in the transcriptome comparison of male-sterile and fertile lines [45–48]. Consistent with these results, we anticipate that the DAH3615-MS, which lacks pollen and exhibits small-sized stamen, is deficient in these structural proteins.

Our RNA-seq experiment was based on data from single biological samples; thus, technical validation was required to authenticate those findings. The importance of biological replicates in conducting accurate experiments that take biological variation into account cannot be ignored; however, RNA-seq is frequently been used to pre-screen and narrow the focus of transcriptomic studies, which may then be followed by RT-qPCR. We used RNA-seq analysis to select the most probable candidates for technical validation, then performed RT-qPCR on three biological replicates, and three technical replicates for 33 randomly selected transcripts (14 newly annotated transcripts and 19 DEDEGs). Thirty-two of 33 transcripts were successfully validated (Fig 4 and S2 Fig), and functional annotations of these transcripts were produced. The rest of the newly annotated transcripts discovered by DBA are also strong candidates to be breeding line-specific genes, and further experiments with replication are needed to verify their differential expression.

Among the 18 DEDEGs successfully validated by RT-qPCR, we identified *EXPA9*, which encodes expansin, a cell wall-loosening enzyme located in pollen grains that participates in pollen germination to loosen the cell wall of the stigma and the style, thus helping lignin pollen tube penetration [49, 50]. Additionally, among our validated transcripts were two *TBB1* and one *TBB2* tubulin genes (Fig 4). Alpha-tubulin and beta-tubulin are major components of microtubules, which play roles in pollen development and pollen tube germination [51]. Pyruvate decarboxylase (PDC) transforms pyruvate into acetaldehyde and carbon dioxide [49]. It is abundant in pollen grains and is related to pollen tube germination and growth. *PDC2* is the only functional *PDC* gene in pollen; the *pdc2* knockout mutant had significantly reduced pollen tube growth compared to the wild type. *PDC2* has been suggested as a strong candidate for a role in male sterility in petunia [52].

Our study also revealed that expression of transcripts related to flowering time and organogenesis was biased towards the MF line. A transcript for the phosphatidylinositol/phosphatidylcholine transfer protein SFH3, was highly enriched in the MF line (Fig 4; logFC: −8.3); this protein is reportedly associated with early bolting and early flower formation, giving rise to variation in flower and petal size in *Brassica napus* [53].

Orthologs of some of the genes we identified have been reported to be responsible for inducing male sterility. A transgenic fasciclin-like arabinogalactan protein (*FLA3*)-overexpressing line had reduced stamen filament elongation that was both directly and indirectly associated with male sterility [54]. Based on this report, we anticipate that *FLA5* also has the potential to be involved in male sterility.

Polyubiquitination (catalyzed via UBQ14) regulates various physiological functions such as sexual reproduction. Ubiquitin (Ub) and Ub-conjugated proteins are involved in early anther development in *Nicotiana alata* [55]. The E3 ligase-like protein and the F-box protein are related to male sterility in hybrid rice [56]. Another DEDEG showed similarity to *BAG1*, which encodes a protein with an ubiquitin-like domain and a BAG domain that, like heat shock-induced gene 1, a putative grape BAG protein, promotes the meristematic transition from vegetative to reproductive growth and early flowering [57, 58]. ATPase (encoded by *PMA2*) plays a crucial role in energy release by dephosphorylating ATP to ADP. SPLAYED (*SYD*), a novel SWI/SNF ATPase homolog, interacts with LEAFY, which is a well-known regulator of floral transition in Arabidopsis. As shown by a study of a *syd-2* line, which exhibits male fertility and a reduction in anther dehiscence, SYD is necessary for reproductive and meristem development [59].

Another notable DEDEG was a transcript encoding a protein Stellacyanin (*STEL*), a blue copper protein, which was predominantly expressed in male-fertile watermelon lines. Although, there are no previous reports of a relationship between *STEL* and male sterility or reproductive organ development, our previous RBA results have deduced another blue copper protein to be the most significantly differentially expressed gene in watermelon male-fertile lines compared to male-sterile lines [13]. We therefore conclude that *STEL* could be a novel gene involved in male sterility in watermelon.

The potential links to reproductive development of our candidate genes described above serves to further validate the reliability of DBA, especially in identifying genes that might be helpful for future studies of male sterility in watermelon. Although we have technically validated only 18 of the candidate DEDEGs, the others are also likely to be strong candidates related to male sterility of watermelon–further studies should seek to validate these genes.

To sum up, we carried out DBA to complement RBA on watermelon RNA-seq data. This simultaneous application and comparison of both approaches improved upon RBA alone, as shown by the following results. A total of 855 transcripts were newly discovered using DBA, and 138 DEDEGs were identified as DBA-derived candidate male-sterility genes. The DEDEGs and their technical validation corresponded with RBA results in terms of male-fertility biased expression and genes with analogous functions. Through the functional annotation of our newly annotated transcripts, essential gene functions related to transmembrane transport, homeostasis, stimulus, nucleic acid binding, and Golgi and vesicles were established for watermelon species. Furthermore, our set of 138 putative male sterility-related genes should prove valuable for further watermelon studies. Overall, we conclude that DBA provided a distinct result that could not be discovered using RBA with the current watermelon reference genome. Within the limits of the reference genome of *Citrullus lanatus*, individual application of DBA and RBA can be a valuable tool to complete the transcriptome. The reliable results obtained in this watermelon genome study can be useful for further watermelon transcriptome studies, showing the value of DBA in non-model plant organisms and providing clues to male sterility in watermelon. This integration of DBA and RBA thus contributes to genome study of watermelon as well as to plant male-sterility research.

## Supporting information

S1 Fig Venn diagram showing the comparison between DBA and RBA BLASTX annotations. (PDF)

S2 Fig RT-qPCR results for 14 newly annotated transcripts. (PDF)

S1 File Supplementary tables A–J.
(DOCX)
