# Supplementary figures and images for: De novo-based transcriptome profiling of male-sterile and fertile watermelon lines
Source: PLoS One. 2017 Nov 2;12(11):e0187147. doi: 10.1371/journal.pone.0187147 (PMC5667795; doi:10.1371/journal.pone.0187147)

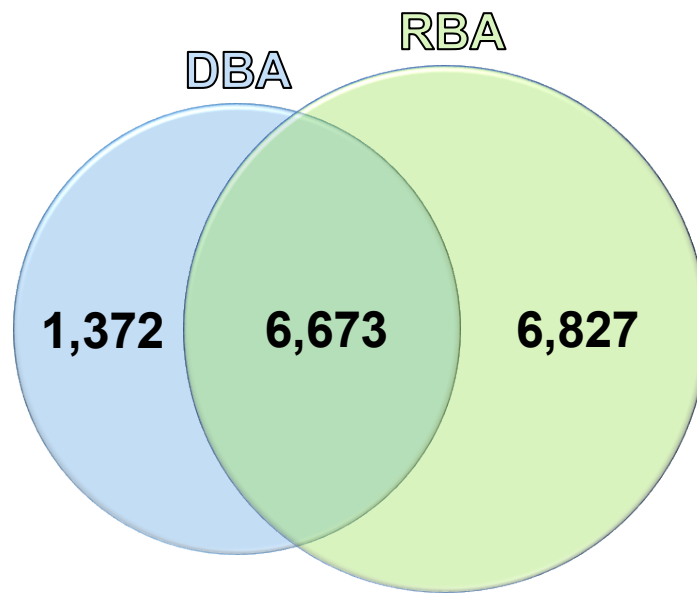

**S1 Fig. Venn diagram showing the comparison between DBA and RBA in BLASTX annotation**

Supplement: S1 Fig — (PDF) [file pone.0187147.s001.pdf]

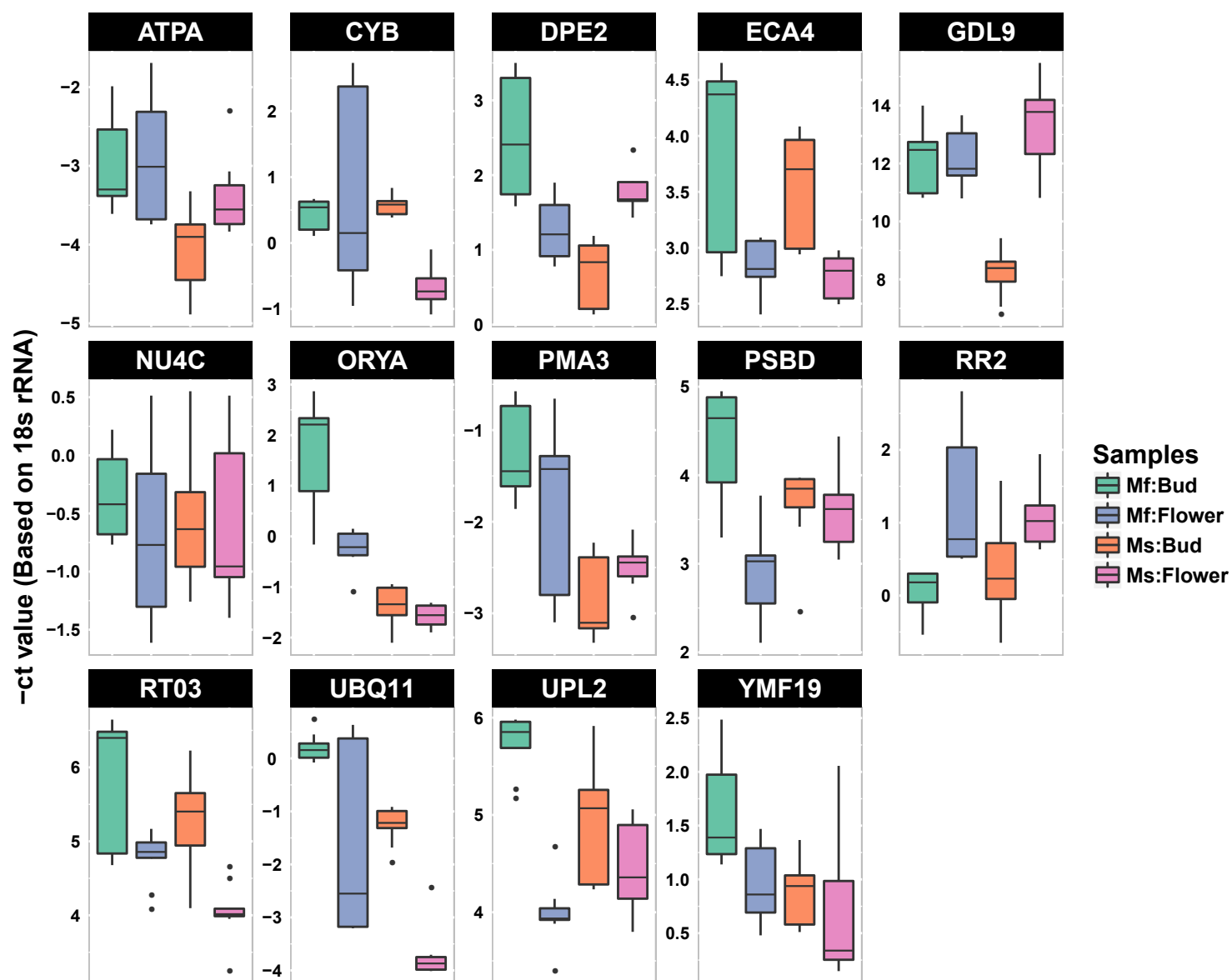

S2 Fig. RT-qPCR results for newly annotated transcripts.

Supplement: S2 Fig — (PDF) [file pone.0187147.s002.pdf]
