# Supplementary material for: De novo-based transcriptome profiling of male-sterile and fertile watermelon lines
Source: PLoS One. 2017 Nov 2;12(11):e0187147. doi: 10.1371/journal.pone.0187147 (PMC5667795; doi:10.1371/journal.pone.0187147)
Supplement: S1 File — Supplementary tables A–J. (DOCX) [file pone.0187147.s003.docx]

***De novo*-based transcriptome profiling of male-sterile and fertile watermelon lines**

Sun-Ju Rhee^1¶^, Taehyung Kwon^2¶^, Minseok Seo^3,4^, Yoon Jung Jang^1^, Tae Yong Sim^1^, Seoae Cho^4^, Sang-Wook Han^1*^, and Gung Pyo Lee^1*^

^1^Department of Integrative Plant Science, Chung-Ang University, Ansung 17546, Republic of Korea

^2^ Department of Agricultural Biotechnology and Research Institute of Agriculture and Life Sciences, Seoul National University, Seoul 151-921, Republic of Korea

^3^Interdisciplinary Program in Bioinformatics, Seoul National University, Kwan-ak St. 599, Kwan-ak Gu, Seoul, South Korea 151-741,Republic of Korea.

^4^CHO&KIM Genomics, C-1008, H Business Park, 26, Beobwon-ro 9-gil, Songpa-gu, Seoul, Republic of Korea

*Corresponding authors

Sang-Wook Han: Tel: +82-31-670-3150, Fax +82-31-675-1969, E-mail: swhan@cau.ac.kr

Gung Pyo Lee: Tel: +82-31-670-3039, Fax +82-31-676-6714, E-mail: gplee@cau.ac.kr

^¶^These authors contributed equally to this work

Email addresses:

SR: [sjrhee825@gmail.com](mailto:sjrhee825@gmail.com)

TK: ted3466@gmail.com

MS: [nijorral@gmail.com](mailto:nijorral@gmail.com)

YJ: [jang.yj781@daum.net](mailto:jang.yj781@daum.net)

TS: [allenst@hanmail.net](mailto:allenst@hanmail.net)

SC: [seoae@cnkgenomics.com](mailto:seoae@cnkgenomics.com)

SH: [swhan@cau.ac.kr](mailto:swhan@cau.ac.kr)

GPL: gplee@cau.ac.kr

**Supplementary Tables**

[Table A. Summary of sequencing results. 3](#_Toc464127402)

[Table B. Summary of *de novo* assembly. 4](#_Toc464127403)

[Table C. Biological process (BP) terms significantly enriched among transcripts newly annotated based on BLASTP. 5](#_Toc464127404)

[Table D. Molecular function (MF) terms significantly enriched among transcripts newly annotated based on BLASTP. 6](#_Toc464127405)

[Table E. Cellular component (CC) terms significantly enriched among transcripts newly annotated based on BLASTP. 8](#_Toc464127406)

[Table F. GO functional terms significantly enriched among 138 DEDEGs. 9](#_Toc464127407)

[Table G. Primer sequences for newly annotated transcripts 11](#_Toc464127408)

[Table H. Gene descriptions of 14 newly annotated transcripts used in RT-qPCR validation 13](#_Toc464127409)

[Table I. Primer sequences for 19 identified DEDEGs 14](#_Toc464127410)

[Table J. Gene descriptions of 19 DEDEGs used in RT-qPCR 16](#_Toc464127411)

Table A. Summary of sequencing results.

| **Sample name** | **Total number of base pairs (nt)** | **Total number of reads** | **GC (%)** | **Q30* (%)** |
| --- | --- | --- | --- | --- |
| **MS_flower** | 3 645 580 719 | 26 501 490 | 42.52 | 79.52 |
| **MS_bud** | 3 607 451 311 | 26 232 292 | 42.48 | 79.89 |
| **MF_flower** | 4 025 519 345 | 29 490 814 | 43.04 | 79.67 |
| **MF_bud** | 3 438 481 342 | 25 299 088 | 42.87 | 79.77 |

MS signifies the DAH3615-MS line, and MF signifies the DAH3615-MF line. Bud refers to the floral bud, and flower refers to the mature flower.

*Percentage of bases having an error probability lower than 0.001

Table B. Summary of *de novo* assembly.

| **Total number of reads** | | 50 581 312 |
| --- | --- | --- |
| **GC percentage** | | 38 |
|  | **Transcripts** | **Genes** |
| Total assembled contigs | 138 811 | 94 496 |
| Total assembled bases (nt) | 152 627 890 | 73 012 104 |
| Average length (nt) | 1100 | 773 |
| N50 contigs (nt) | 2032 | 1327 |

Total transcripts and genes *de novo* assembled using Trinity. ‘Gene’ represents the longest isoform of each transcript.

Table C. Biological process (BP) terms significantly enriched among transcripts newly annotated based on BLASTP.

| **GO identifier** | **Term** | ***P-*Value** |
| --- | --- | --- |
| GO:0055085 | transmembrane transport | 2.40E-^05^ |
| GO:0009628 | response to abiotic stimulus | 2.68E-^05^ |
| GO:0006885 | regulation of pH | 3.70E-^05^ |
| GO:0001101 | response to acid chemical | 3.85E-^05^ |
| GO:0030641 | regulation of cellular pH | 4.50E-^05^ |
| GO:0051453 | regulation of intracellular pH | 4.50E-^05^ |
| GO:0055067 | monovalent inorganic cation homeostasis | 4.59E-^05^ |
| GO:0034220 | ion transmembrane transport | 1.25E-^04^ |
| GO:0006811 | ion transport | 1.38E-^04^ |
| GO:0042592 | homeostatic process | 1.64E-^04^ |
| GO:0050801 | ion homeostasis | 1.73E-^04^ |
| GO:0030004 | cellular monovalent inorganic cation homeostasis | 2.03E-^04^ |
| GO:0055080 | cation homeostasis | 2.26E-^04^ |
| GO:0051606 | detection of stimulus | 3.01E-^04^ |
| GO:0042753 | positive regulation of circadian rhythm | 4.24E-^04^ |
| GO:1901700 | response to oxygen-containing compound | 4.60E-^04^ |
| GO:0098771 | inorganic ion homeostasis | 5.20E-^04^ |
| GO:0071214 | cellular response to abiotic stimulus | 7.78E-^04^ |

Enrichment test *P*-value ≤ 0.001.

Table D. Molecular function (MF) terms significantly enriched among transcripts newly annotated based on BLASTP.

| **GO identifier** | **Term** | ***P-*Value** |
| --- | --- | --- |
| GO:0042626 | ATPase activity, coupled to transmembrane movement of substances | 2.67E-^06^ |
| GO:0043492 | ATPase activity, coupled to movement of substances | 2.86E-^06^ |
| GO:0016820 | hydrolase activity, acting on acid anhydrides, catalyzing transmembrane movement of substances | 3.76E-^06^ |
| GO:0015405 | P-P-bond-hydrolysis-driven transmembrane transporter activity | 4.76E-^06^ |
| GO:0015399 | primary active transmembrane transporter activity | 5.06E-^06^ |
| GO:0005524 | ATP binding | 6.29E-^06^ |
| GO:0035639 | purine ribonucleoside triphosphate binding | 1.45E-^05^ |
| GO:0000166 | nucleotide binding | 2.49E-^05^ |
| GO:1901265 | nucleoside phosphate binding | 2.49E-^05^ |
| GO:0032559 | adenyl ribonucleotide binding | 2.69E-^05^ |
| GO:0030554 | adenyl nucleotide binding | 2.85E-^05^ |
| GO:0022804 | active transmembrane transporter activity | 3.67E-^05^ |
| GO:0042623 | ATPase activity, coupled | 4.29E-^05^ |
| GO:0097367 | carbohydrate derivative binding | 4.33E-^05^ |
| GO:0001882 | nucleoside binding | 4.53E-^05^ |
| GO:0032555 | purine ribonucleotide binding | 5.48E-^05^ |
| GO:0017076 | purine nucleotide binding | 5.69E-^05^ |
| GO:0032553 | ribonucleotide binding | 5.92E-^05^ |
| GO:0036094 | small molecule binding | 6.07E-^05^ |
| GO:0001883 | purine nucleoside binding | 6.54E-^05^ |
| GO:0032550 | purine ribonucleoside binding | 6.54E-^05^ |
| GO:0032549 | ribonucleoside binding | 7.21E-^05^ |
| GO:0004674 | protein serine/threonine kinase activity | 5.50E-^04^ |
| GO:0043167 | ion binding | 6.30E-^04^ |
| GO:0022890 | inorganic cation transmembrane transporter activity | 7.09E-^04^ |
| GO:0016462 | pyrophosphatase activity | 7.49E-^04^ |
| GO:0015562 | efflux transmembrane transporter activity | 7.77E-^04^ |
| GO:0016818 | hydrolase activity, acting on acid anhydrides, in phosphorus-containing anhydrides | 8.84E-^04^ |

Enrichment test *P*-value ≤ 0.001

Table E. Cellular component (CC) terms significantly enriched among transcripts newly annotated based on BLASTP.

| **GO identifier** | **Term** | ***P-*Value** |
| --- | --- | --- |
| GO:0005794 | Golgi apparatus | 2.56E-^05^ |
| GO:0005798 | Golgi-associated vesicle | 2.97E-^05^ |
| GO:0016023 | cytoplasmic, membrane-bounded vesicle | 3.14E-^05^ |
| GO:0031988 | membrane-bounded vesicle | 4.50E-^05^ |
| GO:0000139 | Golgi membrane | 5.35E-^05^ |
| GO:0044431 | Golgi apparatus part | 3.30E-^04^ |
| GO:0030135 | coated vesicle | 4.42E-^04^ |
| GO:0044433 | cytoplasmic vesicle part | 4.42E-^04^ |

Enrichment test *P*-value ≤ 0.001.

Table F. GO functional terms significantly enriched among 138 DEDEGs.

| **Category** | **GO identifier** | **Term** | **Genes** | ***P*-Value** |
| --- | --- | --- | --- | --- |
| BP | GO:0030641 | regulation of cellular pH | NHX7_ARATH, AP3BA_ARATH, PMA2_ARATH, VHAA2_ARATH, PMA6_ARATH | 3.40E-^05^ |
| BP | GO:0051453 | regulation of intracellular pH | NHX7_ARATH, AP3BA_ARATH, PMA2_ARATH, VHAA2_ARATH, PMA6_ARATH | 3.40E-^05^ |
| BP | GO:0030004 | cellular monovalent inorganic cation homeostasis | NHX7_ARATH, AP3BA_ARATH, PMA2_ARATH, VHAA2_ARATH, PMA6_ARATH | 8.46E-^05^ |
| BP | GO:0006885 | regulation of pH | NHX7_ARATH, AP3BA_ARATH, PMA2_ARATH, VHAA2_ARATH, PMA6_ARATH | 2.71E-^04^ |
| BP | GO:0055067 | monovalent inorganic cation homeostasis | NHX7_ARATH, AP3BA_ARATH, PMA2_ARATH, VHAA2_ARATH, PMA6_ARATH | 6.79E-^04^ |
| BP | GO:0006811 | ion transport | ATOX1_ARATH, MSL8_ARATH, NHX7_ARATH, PHO19_ARATH, MSL4_ARATH, DTC_ARATH, PMA2_ARATH, VHAA2_ARATH, ACA9_ARATH, KEA2_ARATH, PMA6_ARATH | 3.63E-^03^ |
| BP | GO:0010025 | wax biosynthetic process | KCS6_ARATH, CER3_ARATH, ACLA1_ARATH | 7.74E-^03^ |
| BP | GO:0010166 | wax metabolic process | KCS6_ARATH, CER3_ARATH, ACLA1_ARATH | 8.29E-^03^ |
| BP | GO:0055085 | transmembrane transport | XYLL3_ARATH, NHX7_ARATH, MSL4_ARATH, DTC_ARATH, PMA2_ARATH, UTR3_ARATH, VHAA2_ARATH, ACA9_ARATH, PEX10_ARATH, PMA6_ARATH | 8.37E-^03^ |
| BP | GO:0015992 | proton transport | PMA2_ARATH, VHAA2_ARATH, KEA2_ARATH, PMA6_ARATH | 8.44E-^03^ |
| BP | GO:0006818 | hydrogen transport | PMA2_ARATH, VHAA2_ARATH, KEA2_ARATH, PMA6_ARATH | 8.44E-^03^ |
| BP | GO:0010014 | meristem initiation | ATBH9_ARATH, REV_ARATH, OBE1_ARATH | 9.44E-^03^ |
| MF | GO:0042625 | ATPase coupled ion transmembrane transporter activity | ALA1_ARATH, PMA2_ARATH, VHAA2_ARATH, ACA9_ARATH, PMA6_ARATH | 1.50E-^03^ |
| MF | GO:0015662 | ATPase activity, coupled to transmembrane movement of ions, phosphorylative mechanism | ALA1_ARATH, PMA2_ARATH, ACA9_ARATH, PMA6_ARATH | 1.81E-^03^ |
| MF | GO:0022853 | active ion transmembrane transporter activity | ALA1_ARATH, PMA2_ARATH, VHAA2_ARATH, ACA9_ARATH, PMA6_ARATH | 1.92E-^03^ |
| MF | GO:0004565 | beta-galactosidase activity | BGA12_ARATH, BGAL9_ARATH, BGAL2_ARATH | 3.74E-^03^ |
| MF | GO:0005548 | phospholipid transporter activity | ALA1_ARATH, SFH6_ARATH, SFH3_ARATH | 5.00E-^03^ |
| MF | GO:0015925 | galactosidase activity | BGA12_ARATH, BGAL9_ARATH, BGAL2_ARATH | 5.94E-^03^ |
| MF | GO:0035639 | purine ribonucleoside triphosphate binding | TBB1_ARATH, CRK25_ARATH, MPK15_ARATH, ALA1_ARATH, HSL1_ARATH, MAA3_ARATH, MYO12_ARATH, PMA2_ARATH, FAB1B_ARATH, ITPK2_ARATH, RAH1E_ARATH, ACLA1_ARATH, Y1677_ARATH, GALK1_ARATH, MCM6_ARATH, PUB52_ARATH, ASSY_ARATH, CDPKT_ARATH, ACA9_ARATH, Y5520_ARATH, PI5KA_ARATH, PMA6_ARATH, CDPKL_ARATH | 6.39E-^03^ |
| MF | GO:0015078 | hydrogen ion transmembrane transporter activity | NHX7_ARATH, PMA2_ARATH, VHAA2_ARATH, KEA2_ARATH, PMA6_ARATH | 6.60E-^03^ |
| MF | GO:0005524 | ATP binding | CRK25_ARATH, ALA1_ARATH, MPK15_ARATH, HSL1_ARATH, MAA3_ARATH, MYO12_ARATH, PMA2_ARATH, FAB1B_ARATH, ITPK2_ARATH, ACLA1_ARATH, Y1677_ARATH, GALK1_ARATH, MCM6_ARATH, PUB52_ARATH, ASSY_ARATH, CDPKT_ARATH, ACA9_ARATH, Y5520_ARATH, PI5KA_ARATH, PMA6_ARATH, CDPKL_ARATH | 8.15E-^03^ |
| MF | GO:0015405 | P-P-bond-hydrolysis-driven transmembrane transporter activity | MRAY_ARATH, ALA1_ARATH, PMA2_ARATH, VHAA2_ARATH, ACA9_ARATH, PMA6_ARATH | 8.78E-^03^ |
| MF | GO:0015399 | primary active transmembrane transporter activity | MRAY_ARATH, ALA1_ARATH, PMA2_ARATH, VHAA2_ARATH, ACA9_ARATH, PMA6_ARATH | 8.92E-^03^ |
| MF | GO:0019829 | cation-transporting ATPase activity | PMA2_ARATH, VHAA2_ARATH, ACA9_ARATH, PMA6_ARATH | 9.16E-^03^ |
| MF | GO:0032550 | purine ribonucleoside binding | TBB1_ARATH, CRK25_ARATH, MPK15_ARATH, ALA1_ARATH, HSL1_ARATH, MAA3_ARATH, MYO12_ARATH, PMA2_ARATH, FAB1B_ARATH, ITPK2_ARATH, RAH1E_ARATH, ACLA1_ARATH, Y1677_ARATH, GALK1_ARATH, MCM6_ARATH, PUB52_ARATH, ASSY_ARATH, CDPKT_ARATH, ACA9_ARATH, Y5520_ARATH, PI5KA_ARATH, PMA6_ARATH, CDPKL_ARATH | 9.84E-^03^ |
| MF | GO:0001883 | purine nucleoside binding | TBB1_ARATH, CRK25_ARATH, MPK15_ARATH, ALA1_ARATH, HSL1_ARATH, MAA3_ARATH, MYO12_ARATH, PMA2_ARATH, FAB1B_ARATH, ITPK2_ARATH, RAH1E_ARATH, ACLA1_ARATH, Y1677_ARATH, GALK1_ARATH, MCM6_ARATH, PUB52_ARATH, ASSY_ARATH, CDPKT_ARATH, ACA9_ARATH, Y5520_ARATH, PI5KA_ARATH, PMA6_ARATH, CDPKL_ARATH | 9.84E-^03^ |
| CC | GO:0005773 | vacuole | TBB1_ARATH, BGA12_ARATH, DTC_ARATH, PMA2_ARATH, PPA26_ARATH, FAB1B_ARATH, VHAA2_ARATH, VA713_ARATH, AT18F_ARATH, CML5_ARATH, HEAT1_ARATH, BGAL9_ARATH, DUS3L_ARATH, UBQ14_ARATH, BGAL2_ARATH | 5.46E-^03^ |
| CC | GO:0031301 | integral component of organelle membrane | CML5_ARATH, PLRX3_ARATH, UTR3_ARATH, PEX10_ARATH | 6.54E-^03^ |
| CC | GO:0031300 | intrinsic component of organelle membrane | CML5_ARATH, PLRX3_ARATH, UTR3_ARATH, PEX10_ARATH | 7.21E-^03^ |

Enrichment test *P*-value ≤ 0.01.

Table G. Primer sequences for newly annotated transcripts.

| **Contig** | **UniprotKB ID** | **Size** | **Primer** | **Direction** |
| --- | --- | --- | --- | --- |
| c21178_g1 | RT03_PETHY | 119 | GACCCCGTCGTAGTTCTCAA | Forward |
|  |  |  | AAGCGATGCGAGAACGTATT | Backward |
| c24422_g1 | ORYA_ORYSJ | 129 | GCTCCTTGAAAAAGGCTGTG | Forward |
|  |  |  | CAACACCGTGATCCAGATTG | Backward |
| c25038_g1 | GDL9_ARATH | 125 | CAAGGAACCTCCCCATTTTT | Forward |
|  |  |  | GCAGTGGAAGTCCCAGTGAT | Backward |
| c26468_g1 | PSBD_SOLTU | 103 | AAGTGGCTCATTTCGTACCG | Forward |
|  |  |  | ACTTCCCCACCAGGACCTAC | Backward |
| c26735_g1 | DPE2_ARATH | 111 | GCCCTGCATCCATTGTACTT | Forward |
|  |  |  | ATCCACATCCTTTCCATCCA | Backward |
| c27728_g1 | UPL2_ARATH | 106 | GAACAGGGCAGAAGAAGCAC | Forward |
|  |  |  | AAGCTCGTCATCCTCTTGGA | Backward |
| c28073_g1 | YMF19_HELAN | 137 | CCGTCGACTTATTGGGAAAA | Forward |
|  |  |  | TCCAGGATTGGAAGAAGTGC | Backward |
| c28325_g1 | CYB_ARATH | 101 | GCTTTTGGGGAGCAACTGTA | Forward |
|  |  |  | ATTGTCCACGGAGAAACCAC | Backward |
| c28564_g1 | ECA4_ARATH | 130 | TGTTGCAAATGGAATGCCTA | Forward |
|  |  |  | ATTCCAAGTTTGGCAGCATC | Backward |
| c30204_g4 | RR2_CUCSA | 102 | AAAGGAAAGAGCCGTCAGGT | Forward |
|  |  |  | GTGGAACCCTTGTCTTGGAA | Backward |
| c30669_g1 | NU4C_CUCSA | 110 | TCTCGATGAAATGGGAGGAC | Forward |
|  |  |  | CTGCAACAAAACCGCTCATA | Backward |
| c30720_g2 | ATPA_CUCSA | 109 | TGCCGAATTAGAAGCCTTTG | Forward |
|  |  |  | TGATTGGGATTGTTTGAGCA | Backward |
| c31499_g1 | UBQ11_ARATH | 107 | AGAAGACGGTCGTACCCTTG | Forward |
|  |  |  | CGGTCAATGTCTTGACGAAA | Backward |
| c32529_g1 | PMA3_NICPL | 106 | AACAACGGGAGCTACAATGG | Forward |
|  |  |  | TGATTGAGCTCGGTGAAGTG | Backward |

Table H. Gene descriptions of 14 newly annotated transcripts used in RT-qPCR validation.

| **Contig** | **UniprotKB ID** | **Gene description** |
| --- | --- | --- |
| c21178_g1 | RT03_PETHY | Ribosomal protein S3, mitochondrial |
| c24422_g1 | ORYA_ORYSJ | Oryzain alpha chain |
| c25038_g1 | GDL9_ARATH | GDSL esterase/lipase At1g28600 |
| c26468_g1 | PSBD_SOLTU | Photosystem II D2 protein |
| c26735_g1 | DPE2_ARATH | 4-alpha-glucanotransferase DPE2 |
| c27728_g1 | UPL2_ARATH | E3 ubiquitin-protein ligase UPL2 |
| c28073_g1 | YMF19_HELAN | Putative ATP synthase protein YMF19 |
| c28325_g1 | CYB_ARATH | Cytochrome b |
| c28564_g1 | ECA4_ARATH | Calcium-transporting ATPase 4, endoplasmic reticulum-type |
| c30204_g4 | RR2_CUCSA | 30S ribosomal protein S2, chloroplastic |
| c30669_g1 | NU4C_CUCSA | NAD(P)H-quinone oxidoreductase chain 4, chloroplastic |
| c30720_g2 | ATPA_CUCSA | ATP synthase subunit alpha, chloroplastic |
| c31499_g1 | UBQ11_ARATH | Polyubiquitin 11 |
| c32529_g1 | PMA3_NICPL | Plasma membrane ATPase 3 |

Table I. Primer sequences for 19 identified DEDEGs.

| **Contig** | **UniprotKB ID** | **Size** | **Primer** | **Direction** |
| --- | --- | --- | --- | --- |
| c21315_g1 | PME13_ARATH | 106 | CATGCATGCATGTAACGAGG | Forward |
|  |  |  | AGAGCTGGATTGGATCGAGA | Backward |
| c24467_g1 | U86A1_ARATH | 101 | ATGGACGGCAGATGATAACG | Forward |
|  |  |  | CACTTCTCTAACCGCAGCTT | Backward |
| c19315_g1 | TBB2_ELEIN | 115 | GCTTGACTTCACGTTGTTGG | Forward |
|  |  |  | CGCCATGTTCAGAGGAAGAA | Backward |
| c11804_g1 | TBB1_ARATH | 110 | ACTCGGCTCCTTCGGTATAA | Forward |
|  |  |  | TATCAGATCCGGTCCCTACG | Backward |
| c9925_g1 | FLA5_ARATH | 107 | CGGATGATGATGATGCCGAT | Forward |
|  |  |  | TTTAGAAGGAGAACTCGCCG | Backward |
| c23123_g1 | TBB1_ORYSJ | 108 | ATTCCGACCTCCGAAAACTG | Forward |
|  |  |  | CTCTGTATTGCTGCGATCCA | Backward |
| c27749_g1 | PDC2_PEA | 109 | TTACTCGGCACAGATTGAGC | Forward |
|  |  |  | AGAAGTTGAAGCTGCCCAAA | Backward |
| c29764_g1 | EXPA9_ARATH | 116 | GCCGAACTACAACCTTGCTA | Forward |
|  |  |  | GAACAATGCCAGCCTTGAAC | Backward |
| c26591_g1 | SFH3_ARATH | 117 | TCTCCCACCCTTTTGATCCT | Forward |
|  |  |  | CTTCGTTGGCACCTTTGTTC | Backward |
| c26250_g1 | COL3_ARATH | 120 | GAGCACGAAGGAGAAGAAGG | Forward |
|  |  |  | GTGTCCTATGGCTCCAGTTG | Backward |
| c21566_g1 | RLF9_ARATH | 113 | TTCTCGTCACGATCGAAAAA | Forward |
|  |  |  | AGCCCAAAACATCCACAACT | Backward |
| c21122_g2 | UBQ14_ARATH | 102 | GGTGGAATGCCCTCCTTATC | Forward |
|  |  |  | TGTGAAAACCTTGACTGGCA | Backward |
| c21122_g1 | UBQ14_ARATH | 117 | TCGCCTTCACATTGTCGATT | Forward |
|  |  |  | CTCTCCATTTGGTGCTTCGA | Backward |
| c27243_g1 | PI5KA_ARATH | 100 | CCTACCTGACCCTCAAATGC | Forward |
|  |  |  | AGTGGGGGAGAGATCAACAA | Backward |
| c27384_g1 | P2C73_ORYSJ | 102 | TCAACCTGAAAATGGGGTGG | Forward |
|  |  |  | TCAAAAGCAGCCTCCCATTT | Backward |
| c23542_g1 | STEL_TOXVR | 114 | ATGGTTGGAGAGTCCCTGAA | Forward |
|  |  |  | CTAGGTTGTTGGCACCTGAA | Backward |
| c26797_g1 | E13A_SOYBN | 105 | ATTGCCAGTCATGATGTCCC | Forward |
|  |  |  | TCTTTAGTGGCCAATGCTCC | Backward |
| c17295_g1 | BAG1_ARATH | 102 | CTCCTTCGCGATGATTTCCT | Forward |
|  |  |  | GAGAGGTTGAGGAGAGAGCT | Backward |
| c16545_g1 | PMA2_ARATH | 114 | GATCATCCCTATCGCGATCG | Forward |
|  |  |  | TTCTTTGGAAAGGCAGCTCA | Backward |

Table J. Gene descriptions of 19 DEDEGs used in RT-qPCR.

| **Contig** | **UniprotKB ID** | **Gene description** |
| --- | --- | --- |
| c21315_g1 | PME13_ARATH | Probable pectinesterase/pectinesterase inhibitor 13 |
| c24467_g1 | U86A1_ARATH | UDP-glycosyltransferase 86A1 |
| c19315_g1 | TBB2_ELEIN | Tubulin beta-2 chain |
| c11804_g1 | TBB1_ARATH | Tubulin beta-1 chain |
| c9925_g1 | FLA5_ARATH | Fasciclin-like arabinogalactan protein 5 |
| c23123_g1 | TBB1_ORYSJ | Tubulin beta-1 chain |
| c27749_g1 | PDC2_PEA | Pyruvate decarboxylase 2 |
| c29764_g1 | EXPA9_ARATH | Expansin-A9 |
| c26591_g1 | SFH3_ARATH | Phosphatidylinositol/phosphatidylcholine transfer protein SFH3 |
| c26250_g1 | COL3_ARATH | Zinc finger protein CONSTANS-LIKE 3 |
| c21566_g1 | RLF9_ARATH | Protein RALF-like 9 |
| c21122_g2 | UBQ14_ARATH | Polyubiquitin 14 |
| c21122_g1 | UBQ14_ARATH | Polyubiquitin 14 |
| c27243_g1 | PI5KA_ARATH | Phosphatidylinositol 4-phosphate 5-kinase 10 |
| c27384_g1 | P2C73_ORYSJ | Probable protein phosphatase 2C 73 |
| c23542_g1 | STEL_TOXVR | Stellacyanin |
| c26797_g1 | E13A_SOYBN | Glucan endo-1,3-beta-glucosidase |
| c17295_g1 | BAG1_ARATH | BAG family molecular chaperone regulator 1 |
| c16545_g1 | PMA2_ARATH | ATPase 2, plasma membrane-type |
